# Supplementary material for: A viral APOBEC3 antagonist distinguishes HHV-6A from HHV-6B
Source: Nat Commun. 2026 May 1;17:3566. doi: 10.1038/s41467-026-71951-6 (PMC13134964; doi:10.1038/s41467-026-71951-6)
Supplement: Supplementary file 1 — Supplementary Information [file 41467_2026_71951_MOESM1_ESM.pdf]

SUPPLEMENTARY INFORMATION

A viral APOBEC3 antagonist distinguishes HHV-6A from HHV-6B

Jun Arii[a*+], Salma Aktar[a,b,c+], Jing Rin Huang[a], Mansaku Hirai[a], Yoshiki Kawamura[d,e], Hiroki Miura[d], Bochao Wang[a], Satoshi Nagamata[a], Mitsuhiro Nishimura[a], Tetsushi Yoshikawa[d], Reuben S. Harris[b,c] and Yasuko Mori[a]

[a]Division of Clinical Virology, Center for Infectious Diseases, Kobe University Graduate School of Medicine, Kobe, Hyogo, Japan
[b]Department of Biochemistry and Structural Biology, University of Texas San Antonio, San Antonio, TX 78229, USA
[c]Howard Hughes Medical Institute, University of Texas San Antonio, San Antonio, TX 78229, USA
[d]Department of Pediatrics, Fujita Health University School of Medicine, Toyoake, Aichi, Japan
[e]Department of Pediatrics, Fujita Health University Okazaki Medical Center, Okazaki, Aichi Japan

+ The authors contributed equally.

*Address correspondence to:
Dr. Jun Arii
Division of Clinical Virology
Center for Infectious Diseases
Kobe University Graduate School of Medicine
7-5-1 Kusunoki-cho, Chuo-ku, Kobe 650-0017 Japan
Phone: 81-78-382-6272
Fax: 81-78-382-6879
E-mail: jarii@med.kobe-u.ac.jp

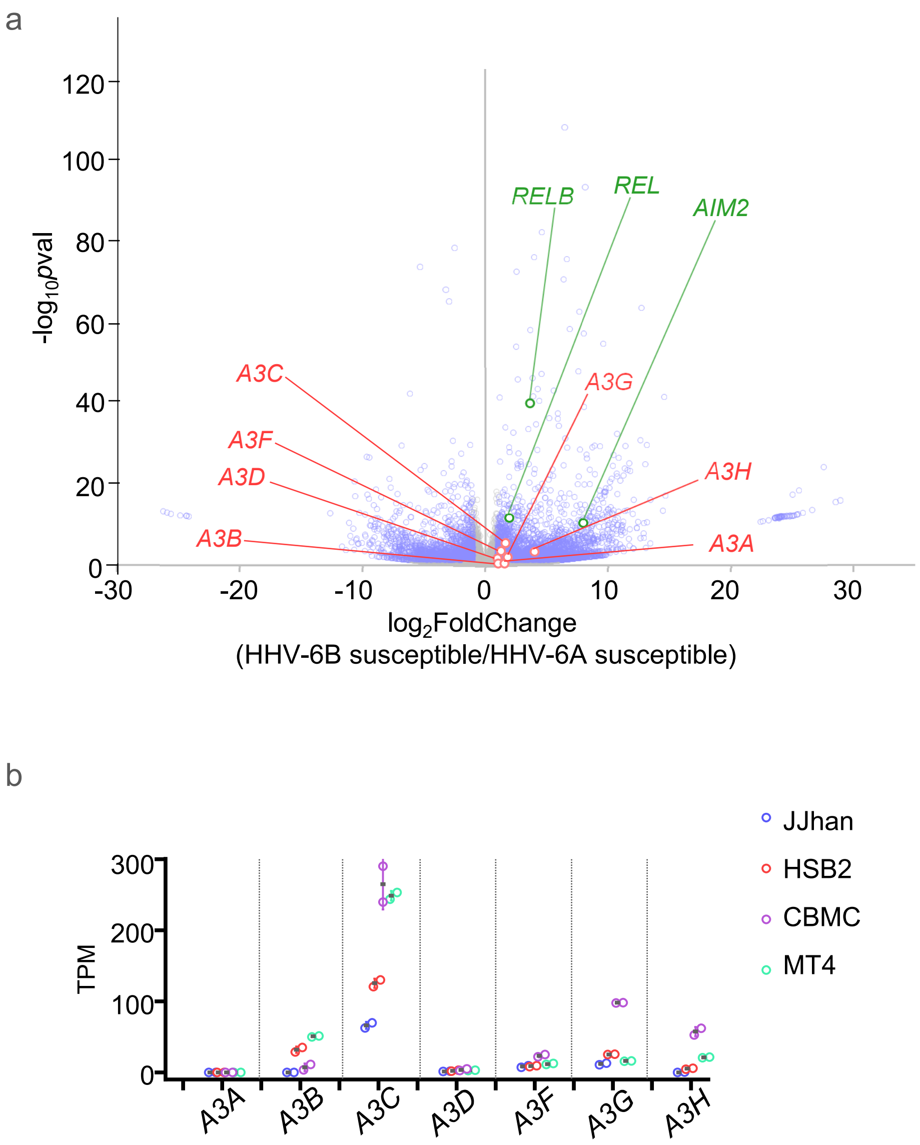

Supplementary Fig. 1 Expression of APOBEC3 family genes in HHV-6B-susceptible cells (a) Volcano plot of DEGs comparing HHV-6B-susceptible and HHV-6A-susceptible cells. APOBEC3 family genes are labeled in pink; other known restriction factors (e.g., *REL*, *RELB*, *AIM2*) are highlighted in green; remaining innate-immune DEGs are shown in blue, and non-DEGs in gray. Differential expression was analyzed using DESeq2 with the Wald test. Because this analysis was exploratory, genes were displayed using thresholds of $|\log_2$ fold change$| > 1$ and unadjusted $P < 0.05$, without multiple-comparison adjustment. **(b)** Expression levels of the indicated APOBEC3 family members in cord blood mononuclear cells (CBMCs), HSB2, JJhan or MT4 cells measured by RNA-seq. Data are shown from two independent biological replicates (***n*** **= 2** independent experiments). TPM indicates transcripts per million. Source data are provided in the Source Data file.

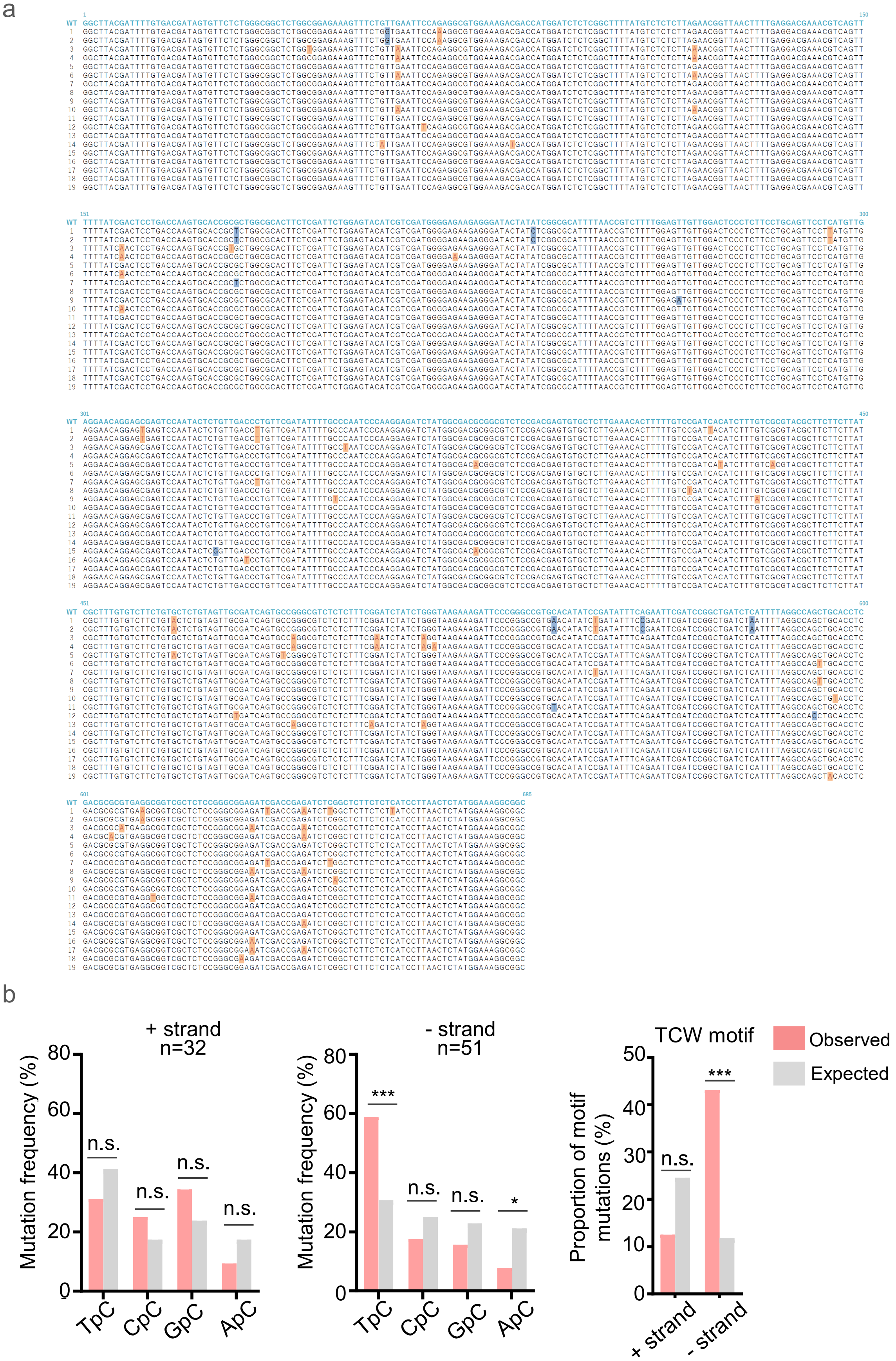
a
b
+ strand
n=32
- strand
n=51
TCW motif
Observed
Expected
Mutation frequency (%)
Proportion of motif mutations (%)
n.s.
***
*
TpC
CpC
GpC
ApC
+ strand
- strand

Supplementary Fig. 2 Mutational patterns of HHV-6A genomes produced in MT4 cells (a) Sanger sequences of cloned *U4* gene segments (685 base pairs) harbouring mutations from MT4 cells infected with HHV-6A, as described in **Fig. 1c**, are shown ($\boldsymbol{n}$ **= 19** mutated clones). The parental wild-type (WT) *U4* sequence is shown in blue (nucleotides +193 to +877 relative to the *U4* start codon). C/G-to-T/A substitutions are highlighted in orange; other types of mutations are highlighted in blue. Clone numbers are shown on the left. All clones containing mutations were detected only once. **(b)** Context-dependent mutation frequencies for C/G-to-T/A substitutions detected in *U4* clones from HHV-6A–infected MT4 cells. For each strand (+, $\boldsymbol{n}$ **= 32** substitutions; −, $\boldsymbol{n}$ **= 51** substitutions), the observed fractions of mutations occurring in NpC contexts (TpC, CpC, GpC, ApC) are shown (pink) together with the expected fractions based on motif availability in the *U4* reference sequence (grey). Right, the proportion of TCW (W = A or T) within TC-context mutations on each strand (observed vs expected). Significance was evaluated by two-sided binomial tests against the expected fraction; q values for NpC context comparisons were adjusted by the Benjamini–Hochberg method within each strand (n.s., not significant; * p or $q < 0.05$; *** p or $q < 0.001$). Exact P values and detailed statistics for **(b)** are provided in the Source Data file. Source data are provided in the Source Data file.

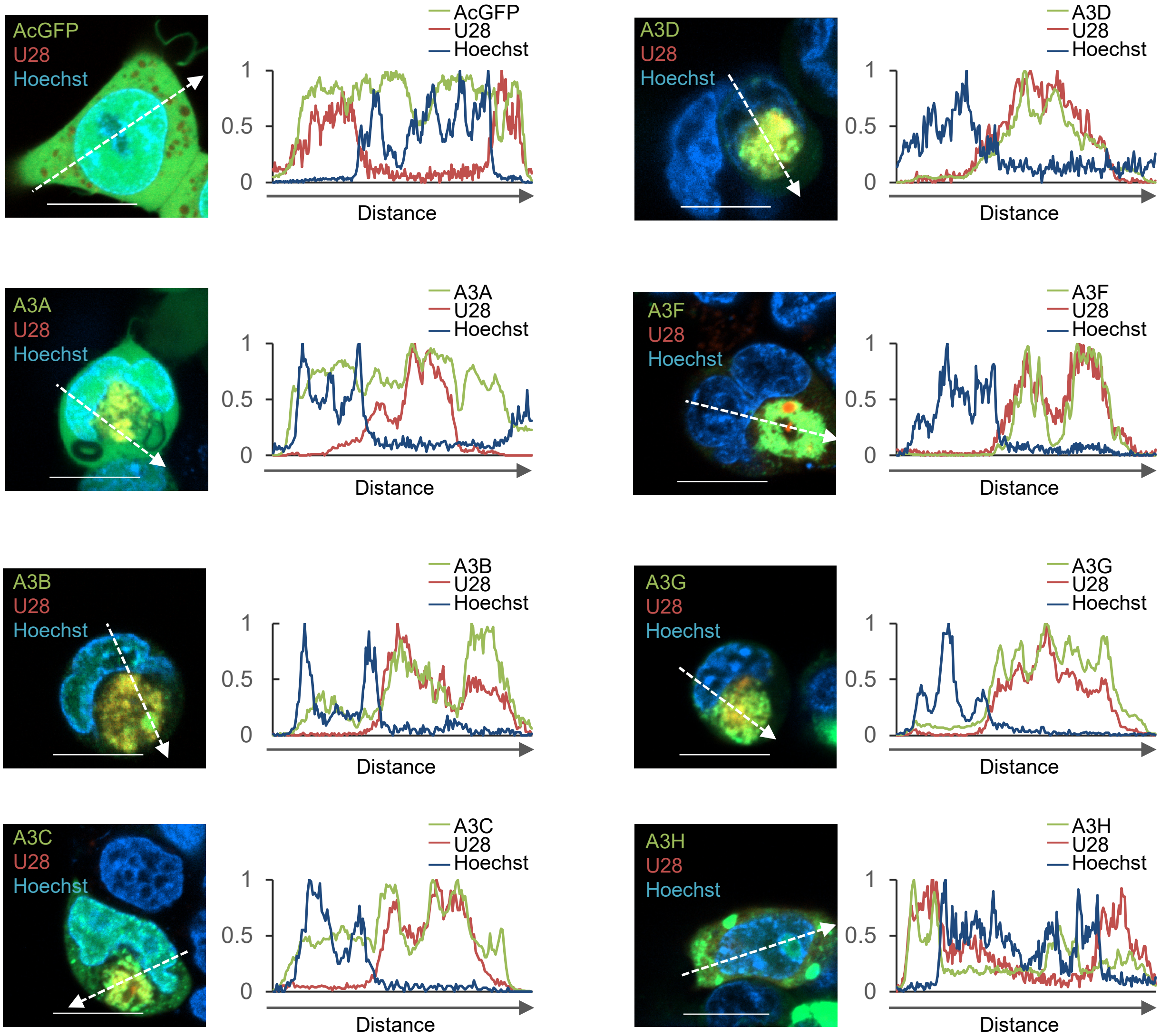

Supplementary Fig. 3 Colocalization of HHV-6B U28 with A3 proteins in HEK293T cells Immunofluorescence microscopy images of HEK293T cells described in **Fig. 3a** are shown. Fluorescence line scans along the dotted lines in the images are displayed at the far right of each panel. Scale bars, 10 µm. Images are representative of three independent experiments. Source data for the fluorescence line scans are provided in the Source Data file.

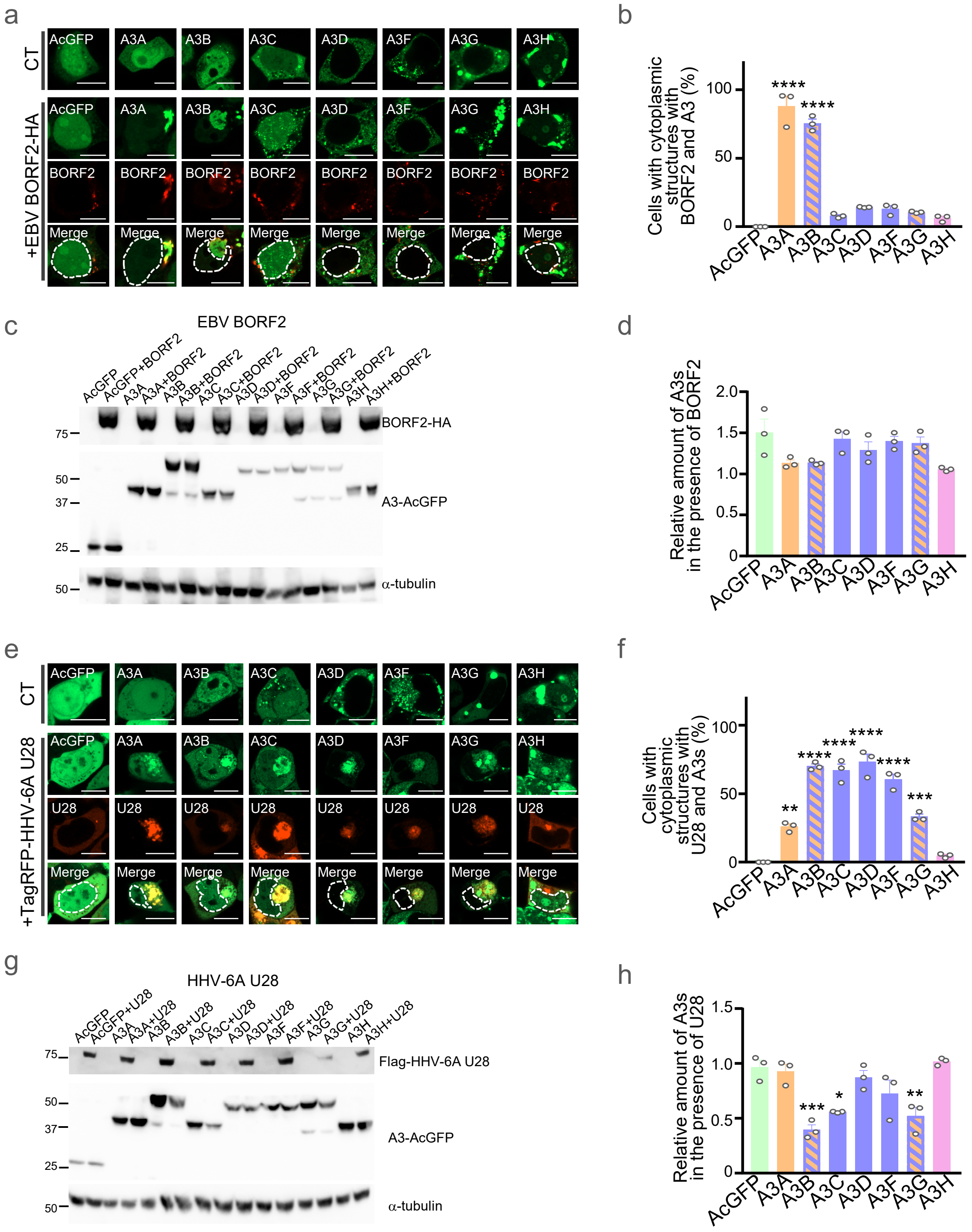

Supplementary Fig. 4 Effects of HHV-6A U28 and Epstein–Barr virus (EBV) BORF2 on localization and stability of A3 proteins (a, e) Fluorescence microscopy images of HEK293T cells expressing individual A3-AcGFP and either EBV BORF2-HA or control vector (CT) (**a**, stained with anti-HA antibody) or TagRFP-HHV-6A U28 or CT (**e**). Dotted lines indicate nuclear membranes. Scale bars, 10 μm. Images are representative of three independent experiments. **(b, f)** Percentage of cells with cytoplasmic A3-AcGFP and either EBV BORF2-HA (**b**) or TagRFP-HHV-6A U28 **(f)**, in the experiments shown in panels **(a)** and **(e)**, respectively. For each condition, 30–100 cells were analyzed per independent experiment. Data are shown as mean ± s.e.m. from three independent biological replicates (***n*** **= 3**). Statistical significance was assessed by one-way ANOVA followed by Tukey's multiple-comparisons test; comparisons shown are relative to A3H-AcGFP in **(b)** and **(f)**; **, $P < 0.01$; ***, $P < 0.001$; ****, $P < 0.0001$. **(c, g)** Immunoblots of lysates from HEK293T cells expressing individual A3-AcGFP and either EBV BORF2-HA or Flag-HHV-6A U28. Immunoblot labels indicate molecular weight (kDa). Images are representative of three independent experiments. **(d, h)** Intensities of A3-AcGFP in the presence of EBV BORF2 or HHV-6A U28 in each lane in panels **(c)** and **(g)**, respectively, normalized to α-tubulin, and divided by those without EBV BORF2 or HHV-6A U28. Data are shown as mean ± s.e.m. from three independent biological replicates (***n*** **= 3**). Statistical significance was assessed by one-way ANOVA followed by Tukey's multiple-comparisons test; comparisons shown are relative to AcGFP; *, $P < 0.05$; **, $P < 0.01$; ***, $P < 0.001$. Exact P values and detailed statistics for **(b)**, **(d)**, **(f)**, and **(h)** are provided in the Source Data file. Source data are provided in the Source Data file.

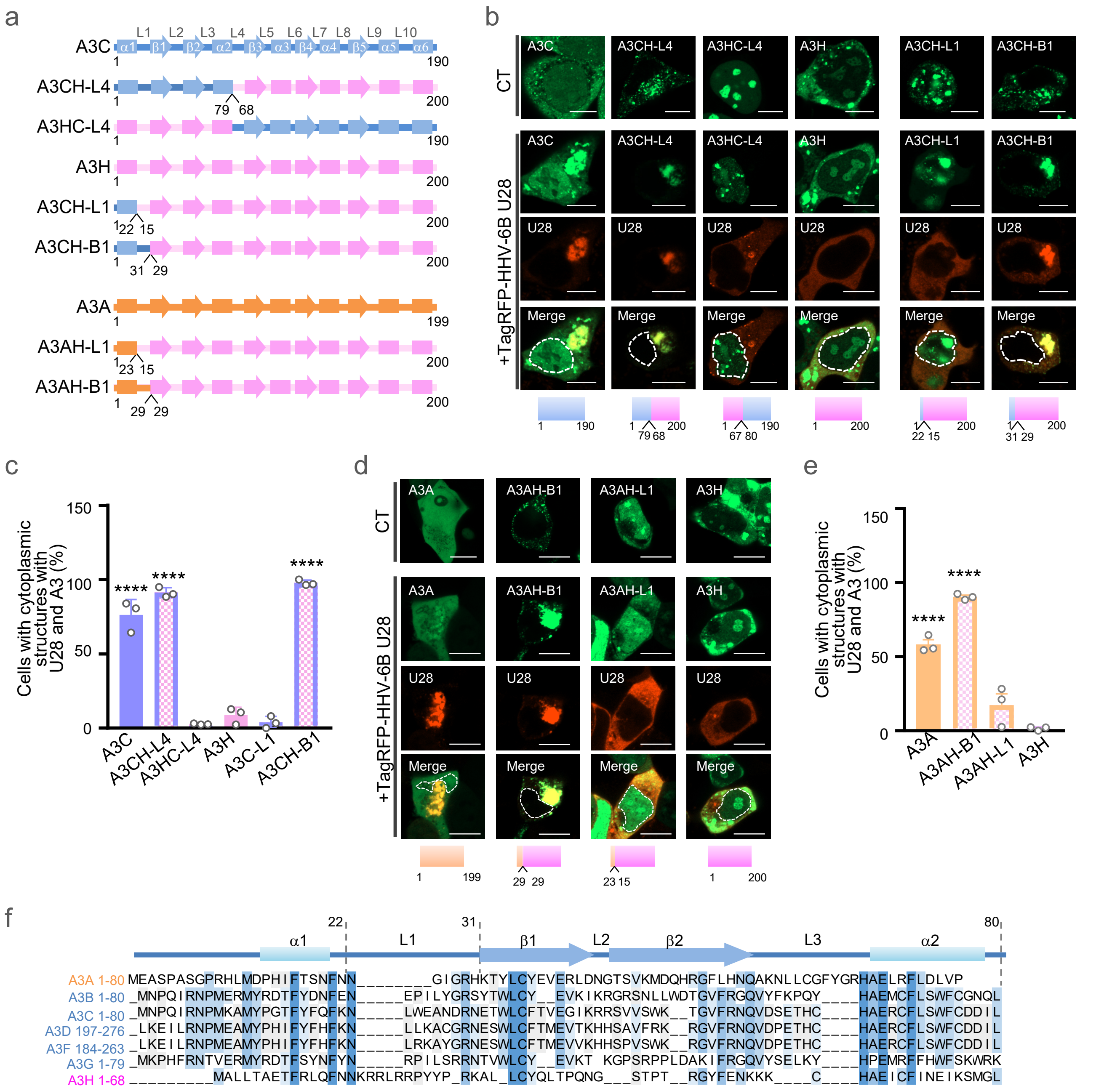

Supplementary Fig. 5 N-terminal regions of A3C and A3A mediate redistribution by HHV-6B U28 (a) Schematic of secondary structures of the A3C–A3H and A3A–A3H chimeras. α, β, and L denote α-helix, β-sheet, and loop, respectively. **(b, d)** Fluorescence microscopy image of HEK293T cells expressing AcGFP-tagged A3C–A3H **(b)** or A3A–A3H **(d)** chimeras together with control vector (CT) or TagRFP–HHV-6B U28. Scale bars, 20 μm. Dotted lines indicate nuclear membranes. Images are representative of three independent experiments. **(c, e)** Percentage of cells showing cytoplasmic A3–AcGFP together with TagRFP–HHV-6B U28 in the experiments shown in **(b)** and **(d)**, respectively. For each condition, 30–100 cells were analyzed per independent experiment. Data are shown as mean ± s.e.m. from three independent biological replicates (***n*** = **3**). Statistical significance was assessed by one-way ANOVA followed by Tukey's multiple-comparisons test; comparisons shown are relative to A3H-AcGFP in **(c)** and **(e)**; ****, $P < 0.0001$. **(f)** Schematic of the secondary structure of the N-terminal region of the Z domain. Amino-acid sequences corresponding to the N-terminal region (aa 1–80 in A3C) of seven APOBEC3 proteins are shown. Exact P values and detailed statistics for **(c)** and **(e)**, and source data are provided in the Source Data file.

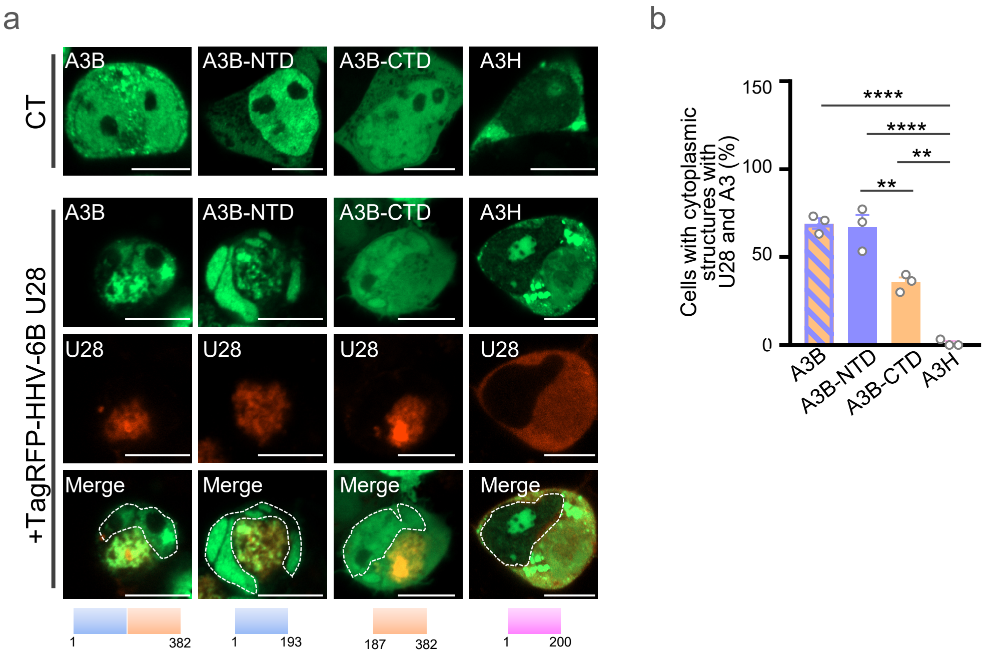

Supplementary Fig. 6 Domain sufficiency in A3B for HHV-6B U28–dependent redistribution (**a**) Fluorescence microscopy images of HEK293T cells co-expressing AcGFP-tagged A3B, A3B-NTD, A3B-CTD or A3H together with control vector (CT) or TagRFP–HHV-6B U28. Scale bars, 10 µm. Dotted lines indicate nuclear membranes. Images are representative of three independent experiments. (**b**) Percentage of cells showing cytoplasmic A3–AcGFP together with TagRFP–HHV-6B U28 in the experiments shown in **(a)**. For each condition, 30–40 cells were analyzed per independent experiment. Data are shown as mean ± s.e.m. from three independent biological replicates (***n*** = **3**). Statistical significance was assessed by one-way ANOVA followed by Tukey's multiple-comparisons test; **, $P < 0.01$; ****, $P < 0.0001$. Exact *P* values and detailed statistics for **(b)** are provided in the Source Data file. Source data are provided in the Source Data file.

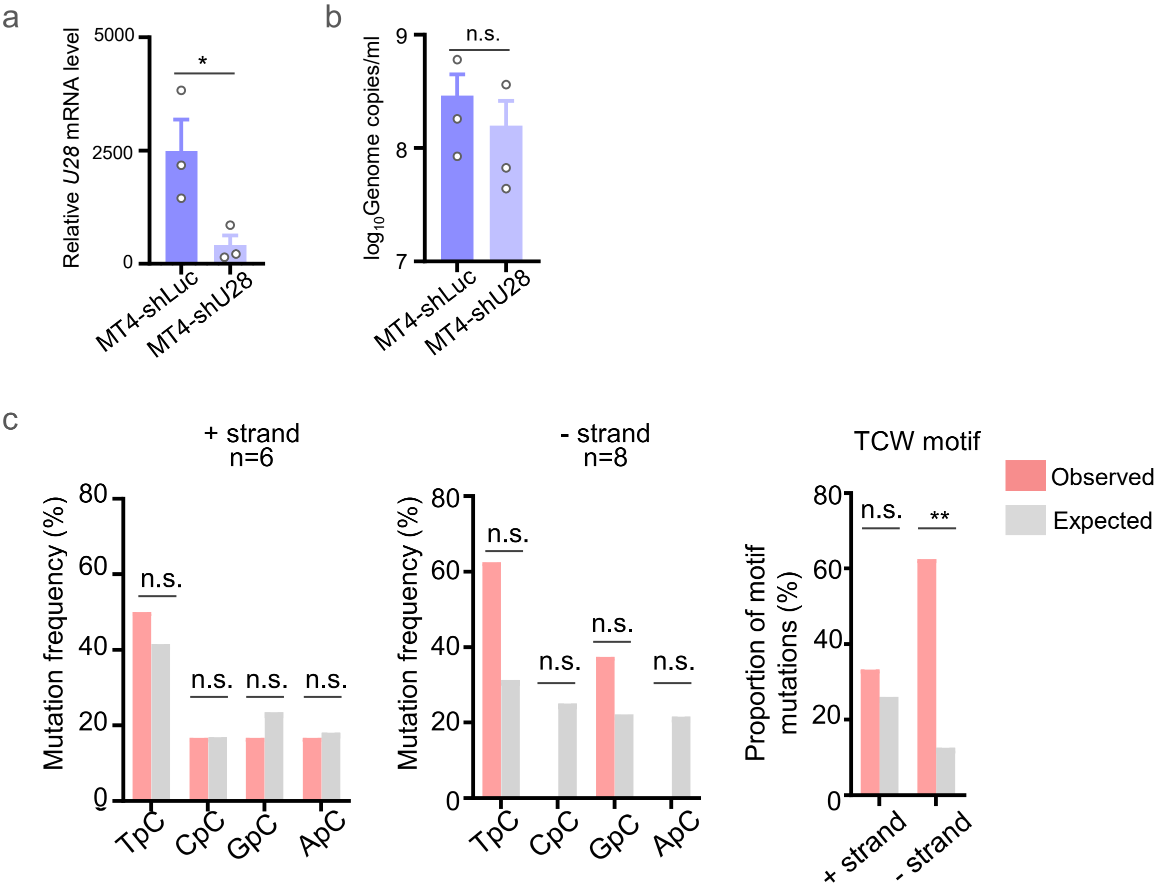

Supplementary Fig. 7 Effects of *U28* knockdown on *U28* mRNA levels and HHV-6B replication (a) *U28* mRNA levels measured by RT-qPCR in MT4 cells expressing shRNA targeting luciferase or *U28* after infection with HHV-6B, normalized to β-actin. Data are shown as mean ± s.e.m. from three independent biological replicates (***n*** **= 3**). Statistical significance was assessed by unpaired two-tailed Student's *t*-test; *, $P < 0.05$. **(b)** Viral yields in the supernatant of MT4 cells expressing the indicated shRNAs and infected with HHV-6B for 72 h. Data are shown as mean ± s.e.m. from three independent biological replicates (***n*** **= 3**). Statistical significance was assessed by unpaired two-tailed Student's t-test; n.s., not significant. **(c)** Context-dependent mutation frequencies for G-to-A/C-to-T substitutions detected in *U4* clones from HHV-6B–infected MT4-shU28 cells. For each strand (+, ***n*** **= 6** substitutions; −, ***n*** **= 8** substitutions), the observed fractions of mutations occurring in NpC contexts (TpC, CpC, GpC, ApC) are shown (pink) together with the expected fractions based on motif availability in the *U4* reference sequence (grey). Right, the proportion of TCW (W = A or T) within TC-context mutations on each strand (observed vs expected). Significance was evaluated by two-sided binomial tests against the expected fraction; *q* values for NpC context comparisons were adjusted by the Benjamini–Hochberg method within each strand (n.s., not significant; ** *p* or $q < 0.01$). Exact *P* values and detailed statistics for **(a–c)** are provided in the Source Data file. Source data are provided in the Source Data file.

a

	Age (years)	Sex	Stem cell type
Patient 1	≥60	M	cord blood
Patient 2	40–59	F	unrelated bone marrow from unrelated donor
Patient 3	≥60	F	unrelated bone marrow from unrelated donor
Patient 4	40–59	M	unrelated peripheral blood
Patient 5	40–59	M	cord blood
Patient 6	≥60	M	cord blood
Patient 7	40–59	M	cord blood
Patient 8	20–39	M	related peripheral blood stem cell
Patient 9	40–59	F	related peripheral blood stem cell

b

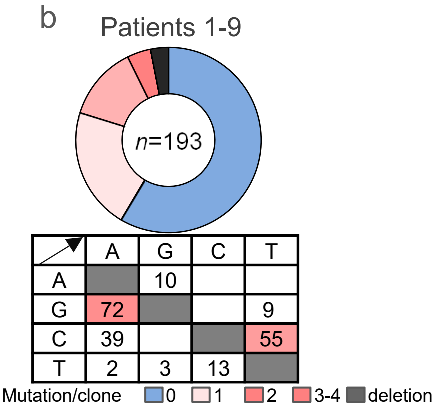

c

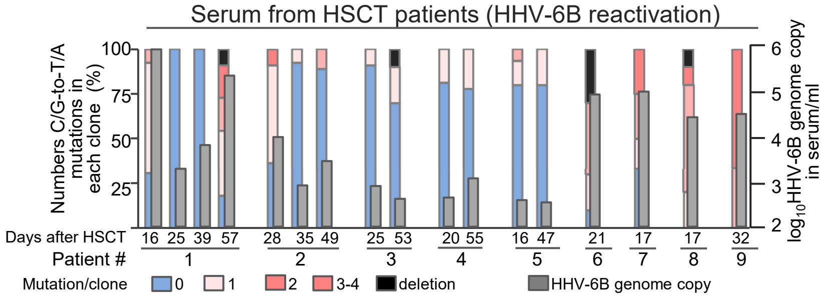

d

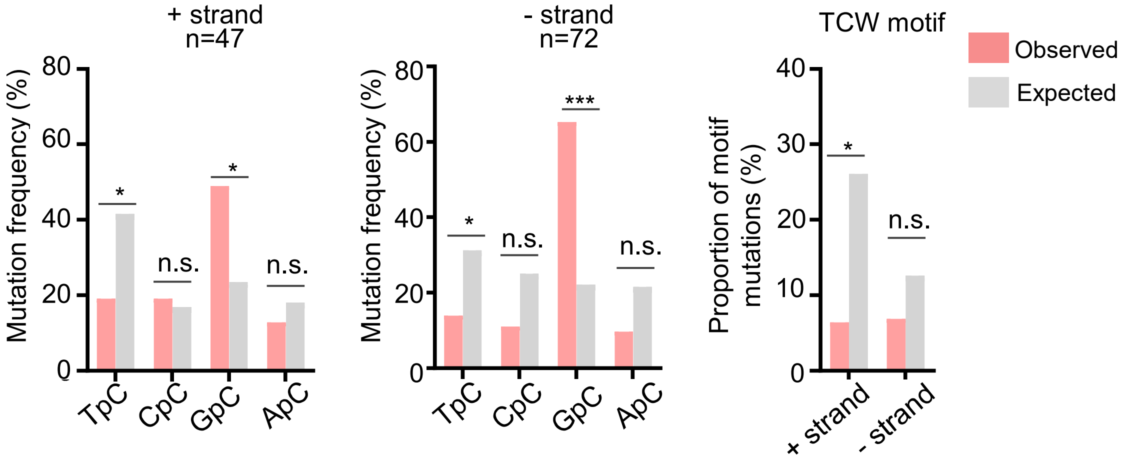

e

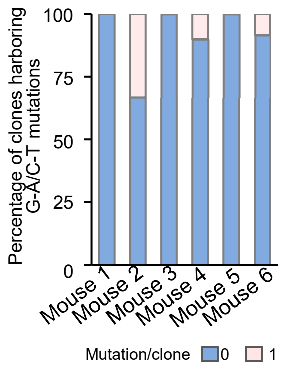

f

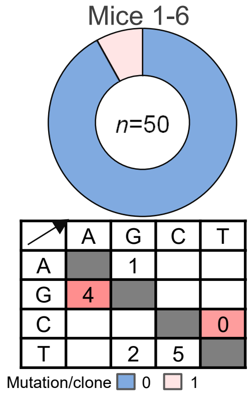

Supplementary Fig. 8 Mutational patterns of HHV-6B genomes in vivo (a) Summary of enrolled patients 1–9. **(b)** Pie chart showing the number of C/G-to-T/A mutations observed in sequence analysis described in **Fig. 7a** compared to the HHV-6B HST strain. The mutation matrix of all types of events is shown below. **(c)** Stacked bar graphs showing HHV-6B genome copy numbers along with the percentage of clones with the indicated number of C/G-to-T/A mutations in the *U4* gene segment in serum from nine patients collected at the indicated days after transplant, as described in **Fig. 7a**. **(d)** Context-dependent mutation frequencies for G-to-A/C-to-T substitutions detected in the *U4* gene segment from serum samples of nine patients. For each strand (+, $\boldsymbol{n}$ **= 47** substitutions; −, $\boldsymbol{n}$ **= 72** substitutions), the observed fractions of mutations occurring in NpC contexts (TpC, CpC, GpC, ApC) are shown (pink) together with the expected fractions based on motif availability in the *U4* reference sequence (grey). Right, the proportion of TCW (W = A or T) within TC-context mutations on each strand (observed vs expected). Significance was evaluated by two-sided binomial tests against the expected fraction; q values for NpC context comparisons were adjusted by the Benjamini–Hochberg method within each strand (n.s., not significant; * p or $q < 0.05$; *** p or $q < 0.001$). Exact P values and detailed statistics for **(d)** are provided in the Source Data file. **(e)** Stacked bar graphs showing the percentage of clones with the indicated number of C/G-to-T/A mutations observed in Sanger sequences of cloned *U4* gene segments from viral DNA collected from the spleens of six mice 6–9 days after HHV-6B infection. **(f)** Pie chart showing the number of C/G-to-T/A mutations observed in sequence analysis described in **(e)** compared to the HHV-6B HST strain. Mutation matrices of all types of events are shown below the chart. Source data for **(b–f)** are provided in the Source Data file.

a

	Age (years)	Sex	Clinical status
Patient 10	0–19	M	acute encephalopathy
Patient 11	0–19	M	acute encephalopathy
Patient 12	0–19	F	acute encephalopathy with biphasic seizures and late reduced diffusion (late seizure)
Patient 13	0–19	M	X-SCID + ciHHV-6A/ HSCT

b

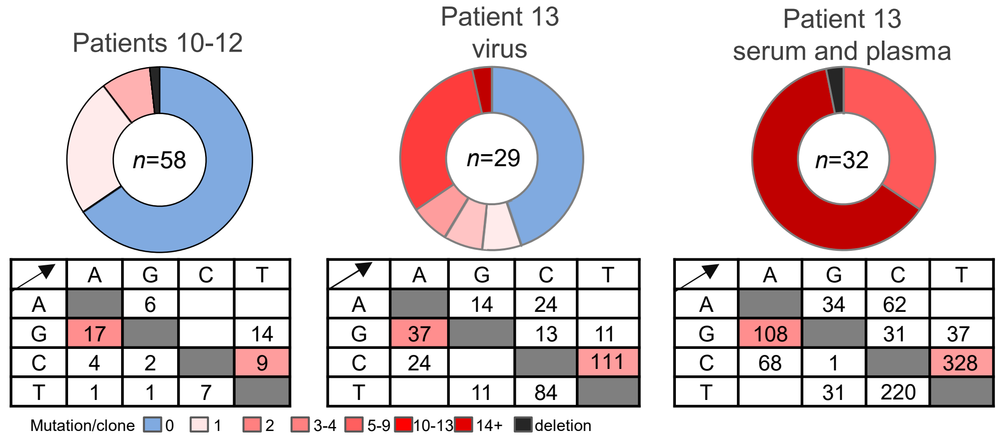

c

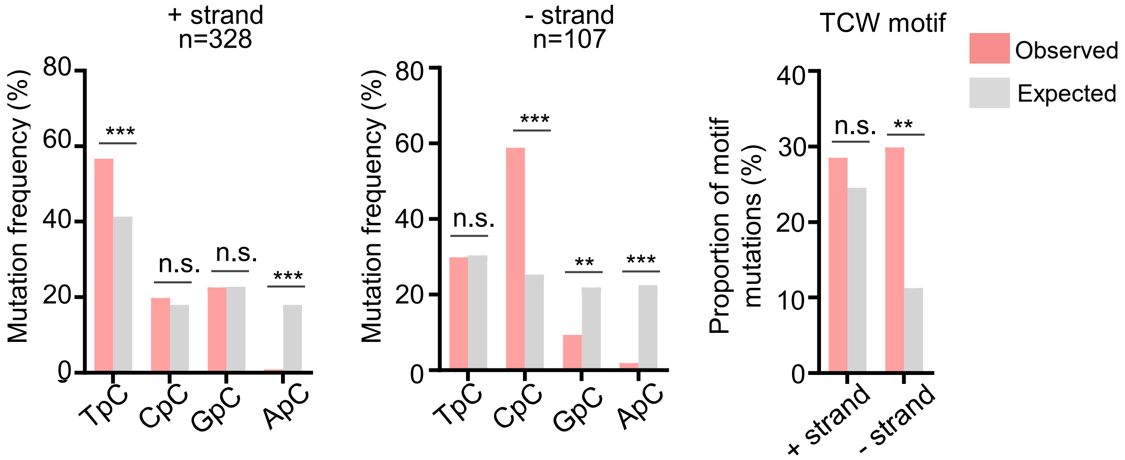

Supplementary Fig. 9 Mutational patterns of HHV-6A and HHV-6B genomes in vivo (a) Summary of enrolled patients 10–13. **(b)** Pie charts showing the number of C/G-to-T/A mutations observed in sequence analysis described in **Fig. 7c** and **Fig. 8a**, compared to the HHV-6B HST strain or iciHHV-6A from patient 13, respectively. Mutation matrices of all types of events are shown below each chart. **(c)** Context-dependent mutation frequencies for G-to-A/C-to-T substitutions pooled across all *U4* clones analyzed. For each strand (+, $\boldsymbol{n} = \mathbf{328}$ substitutions; −, $\boldsymbol{n} = \mathbf{107}$ substitutions), the observed fractions of mutations occurring in NpC contexts (TpC, CpC, GpC, ApC) are shown (pink) together with the expected fractions based on motif availability in the *U4* reference sequence (grey). Right, the proportion of TCW (W = A or T) within TC-context mutations on each strand (observed vs expected). Significance was evaluated by two-sided binomial tests against the expected fraction; q values for NpC context comparisons were adjusted by the Benjamini–Hochberg method within each strand (n.s., not significant; ** p or $q < 0.01$; *** p or $q < 0.001$). Exact P values and detailed statistics for **(c)** are provided in the Source Data file. Source data for **(b, c)** are provided in the Source Data file.

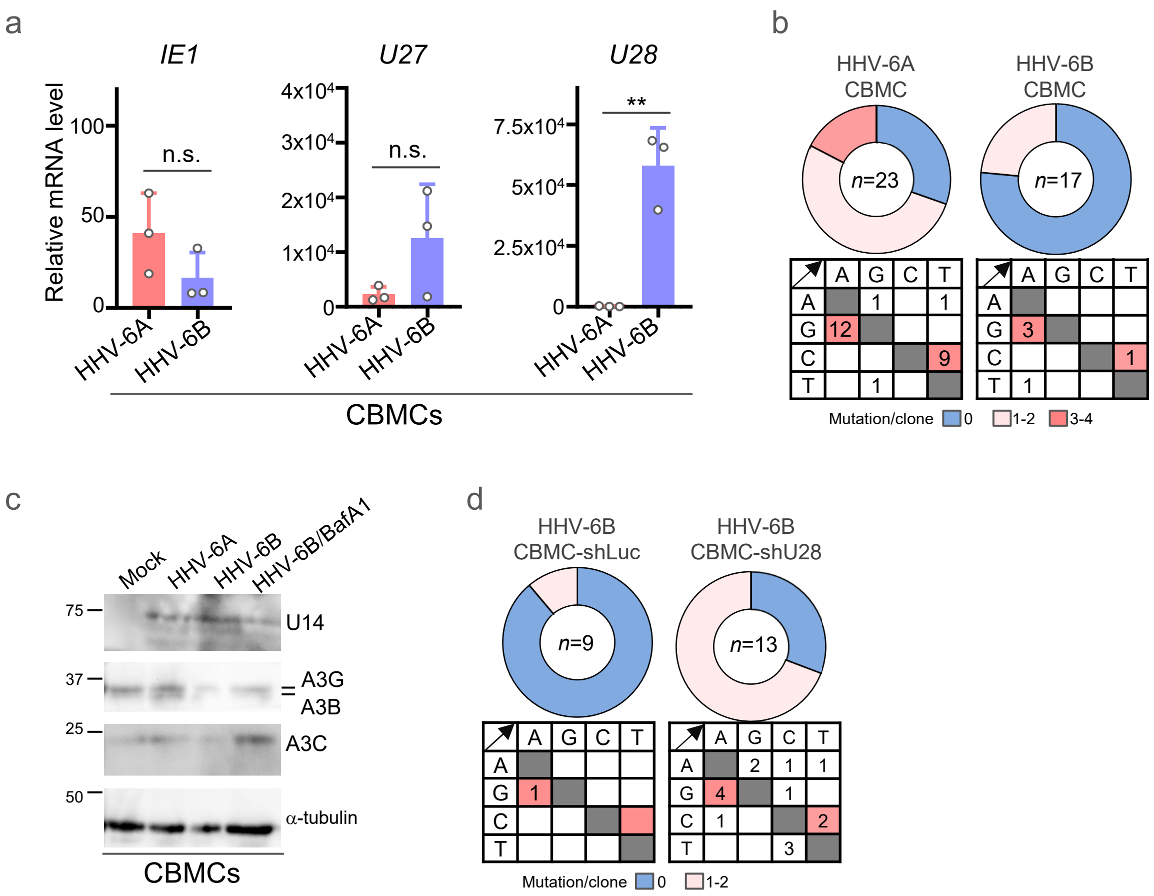

Supplementary Fig. 10 Mutational patterns of HHV-6A and HHV-6B genomes in primary CBMCs (a) mRNA levels of the indicated viral genes by RT-qPCR of CBMCs infected with HHV-6A GS or HHV-6B HST for 48 h, normalized to β-actin. Data are shown as mean ± s.e.m. from three independent biological replicates (***n* = 3**). Statistical significance was assessed by unpaired two-tailed Student's *t*-test; n.s., not significant; **, $P < 0.01$. Exact *P* values and detailed statistics for **(a)** are provided in the Source Data file. **(b)** Pie charts and mutation matrices showing types of mutational events in CBMCs infected with HHV-6A GS or HHV-6B HST, as described in **Fig. 1c**. Non-mutated sequences are depicted in blue; base substitutions with the number of mutations per sequence are depicted in red. **(c)** CBMCs infected with HHV-6A GS or HHV-6B HST for 72 h were mock-treated or treated with BafA1 and analysed by immunoblotting. Immunoblot labels indicate molecular weight (kDa). Images are representative of three independent experiments. **(d)** Pie charts and mutation matrices showing types of mutational events in CBMCs expressing shLuc or shU28 and infected with HHV-6B HST. Source data are provided in the Source Data file.

Supplementary Table 1. Conservation of the *U4* region among HHV-6B strains

Strain name	Accession number	Mutations compared to HST strain
HST	AB021506.1	none
1-ciHHV-6B	KY316046.1	none
2B-9q34.3	KY316045.1	none
4B-11p15.5	KY316044	none
BAN519	KY316043	none
COR264	KY316042	none
CUM082	KY316041.1	none
d37	MW049327.1	none
DER512	KY316040	none
GLA_29221	KY316052.1	none
GLA_34108	KY316051.1	none
GLA_35629	KY316050.1	none
GLA_3986	KY316053.1	none
HAPMAP NA7022	KY316039.1	none
HAPMAP_NA10863	KY316038	none
HGDP00092	KY316037	G254A, A579C
HGDP00813	KY316036	none
HGDP01065	KY316035.1	none
HGDP01077	KY316034.1	none
HHV6B_401027	MW049323.1	A579C
HHV6B_704016	MW049324.1	none
HHV6B_704021	MW049325.1	none
HHV6B_801018	MW049326.1	none
iciHG00245	MG894368.1	none
iciHG00362	MG894369.1	none
iciHG02016	MG894372.1	none
iciHG02301	MG894373.1	none
iciNA19381	MG894375.1	A579C
iciNA19382	MG894376.1	A579C
LEI-ALD	KY316033	none
MOW-F5C	MN242397.1	A542G
ORCA1340	KY316032	none
ORCA1622	KY316031	none
ORCA3835	KY316030	none
Z29	MW536483.1	none
Z29	NC_000898.1	C577A
Z29	AF157706	A579C

Supplementary Table 2. Conservation of the *U4* region among HHV-6A strains

Strain name	Accession number	Mutations compared to GS strain
GS	KC465951.1	none
iciHHV-6A patient 13	this study	G222A, C399T, C592T, C600G, C607T
U1102	NC_001664.4	A237T
AJ	KP257584.1	none
LF5A	MW049322.1	G222A, C579T, C592T, G594A, C600G, C607T
LF3A	MW049321.1	G222A, C579T, C592T, G594A, C600G, C607T
LF2A	MW049320.1	G222A, C579T, C592T, G594A, C600G, C607T
LF1A	MW049319.1	G222A, C579T, C592T, G594A, C600G, C607T
HGDP00628	MW049318.1	G222A, C579T, C592T, G594A, C600G, C607T
HHV6A_506035	MW049317.1	G222A, C579T, C592T, G594A, C600G, C607T
HHV6A_303046	MW049316.1	G222A, C579T, C592T, G594A, C600G, C607T
HHV6A_103091	MW049313.1	G222A, C579T, C592T, G594A, C600G, C607T
7A-17p13.3	KY316048.1	G222A, C579T, C592T, G594A, C600G, C607T
MOW-F1M	MK630134.1	G222A, C579T, C592T, G594A, C600G, C607T
MOW-F1C	MK630133.1	G222A, C579T, C592T, G594A, C600G, C607T
GTEX-1314G_icihhv6b	MH698403.1	G222A, C579T, C592T, G594A, C600G, C607T
GTEX-11DXY	MH698400.1	G222A, C579T, C592T, G594A, C600G, C607T
GLA_25506	KY316054.1	G222A, C579T, C592T, G594A, C600G, C607T
LEI_1501	KT355575.1	G222A, C579T, C592T, G594A, C600G, C607T
GLA_4298	KY316056.1	T384C
Homo sapiens endogenous virus human herpesvirus 6A sequence	KT895199.1	T384C
NA18999	KY316047.1	G222A, C592T, C600G, C607T
3A-10q26.3	KY316049.1	C86T, G222A, G540A, C592T, C600G, C607T

Supplementary Table 3. Oligonucleotide sequences

Target	sequence
HHV-6A genome F	5′-CGCTAGGTTGAGAATGATCGA-3′
HHV-6A genome R	5′-CAAAGCCAAATTATCCAGAGCG-3′
U4 OUT-F	5′-ATTCCCAACCATCCCTATCT-3′
U4 OUT-R	5′-TTGCAGTACACCTTCAAGAT-3′
U4 IN-F	5′-GGCTTACGATTTTGTGACGA-3′
U4 IN-R	5′-GCCGCCTTTCCATAGAGTTA-3′
PO3IE1A	5′-TTCTCCAGATGTGCCAGGGAAATCC-3′
PO3IE1C	5′-CACATTGTTATCGCTTTCACTCTC-3′
casA3B-F	5′-CACCGAGGAGCCCGCGTGACGATCA-3′
casA3B-R	5′-AAACTGATCGTCACGCGGGCTCCTC-3′
casA3C-F	5′-CACCGACCTATGGGAAGCCAACGAT-3′
casA3C-R	5′-AAACATCGTTGGCTTCCCATAGGTC-3′
IE1 RT-F	5′-AATCAATCTTCTGGGTGGGAAGAAAATCCAGCAATGTAATAATTGATGGGTGCAATCG-3′
IE1 RT-R	5′-ACTATTCTCAAGAAGTGGCTCCGGAGAACATTCTCATCACAGACATTCTTTCTTATATCG-3′
U27 RT-F	5′-AAAGCACGTTGTTGACGGTG-3′
U27 RT-R	5′-CCTGTTTTGATGCCAACGCA-3′
U28 RT-F	5′-ATGTGCCTTTGTATTGCGGG-3′
U28 RT-R	5′-ATCGCTTACTTCGTCACCCA-3′
b-actin RT-F	5′-GCACCCAGCACAATGAAGA-3′
b-actin RT-R	5′-CGATCCACACGGAGTACTTG-3′
pInd10-F	5′-CAGAAGGCTCGAGAAGGTATATTGCTGTTGACAGTGAGCG-3′
pInd10-R	5′-CTAAAGTAGCCCCTTGAATTCCGAGGCAGTAGGCA-3′
shLuc oligo	5′-TGCTGTTGACAGTGAGCGCAGGAATTATAATGCTTATCTATAGTGAAGCCACAGATGTATAG ATAAGCATTATAATTCCTATGCCTACTGCCTCGGA-3′
shU28 oligo	5′- TGCTGTTGACAGTGAGCGCGTATAAACACCTTGATTTATCTAGTGAAGCCACAGATGTAGATAA ATCAAGGTGTTTATACATGCCTACTGCCTCGGA -3′
