# Supplementary material for: A viral APOBEC3 antagonist distinguishes HHV-6A from HHV-6B
Source: Nat Commun. 2026 May 1;17:3566. doi: 10.1038/s41467-026-71951-6 (PMC13134964; doi:10.1038/s41467-026-71951-6)
Supplement: Supplementary file 3 — Reporting Summary [file 41467_2026_71951_MOESM3_ESM.pdf]

nature portfolio

Corresponding author(s): Jun Arii

Last updated by author(s): Mar 19, 2026

Reporting Summary

Nature Portfolio wishes to improve the reproducibility of the work that we publish. This form provides structure for consistency and transparency in reporting. For further information on Nature Portfolio policies, see our Editorial Policies and the Editorial Policy Checklist.

Statistics

For all statistical analyses, confirm that the following items are present in the figure legend, table legend, main text, or Methods section.

n/a	Confirmed	
☐	☒	The exact sample size (n) for each experimental group/condition, given as a discrete number and unit of measurement
☐	☒	A statement on whether measurements were taken from distinct samples or whether the same sample was measured repeatedly
☐	☒	The statistical test(s) used AND whether they are one- or two-sided<br>*Only common tests should be described solely by name; describe more complex techniques in the Methods section.*
☐	☒	A description of all covariates tested
☐	☒	A description of any assumptions or corrections, such as tests of normality and adjustment for multiple comparisons
☐	☒	A full description of the statistical parameters including central tendency (e.g. means) or other basic estimates (e.g. regression coefficient) AND variation (e.g. standard deviation) or associated estimates of uncertainty (e.g. confidence intervals)
☐	☒	For null hypothesis testing, the test statistic (e.g. F, t, r) with confidence intervals, effect sizes, degrees of freedom and P value noted<br>*Give P values as exact values whenever suitable.*
☒	☐	For Bayesian analysis, information on the choice of priors and Markov chain Monte Carlo settings
☒	☐	For hierarchical and complex designs, identification of the appropriate level for tests and full reporting of outcomes
☒	☐	Estimates of effect sizes (e.g. Cohen's d, Pearson's r), indicating how they were calculated

Our web collection on statistics for biologists contains articles on many of the points above.

Software and code

Policy information about availability of computer code

Data collection	1. ZEN3.1
Data analysis	1. GraphPad Prism 7 (GraphPad Software)<br>2. MEGA 11 software<br>3. ImageJ software (1.51J8)<br>4. ZEN3.1<br>5. DESeq2 Ver.1.24.0

For manuscripts utilizing custom algorithms or software that are central to the research but not yet described in published literature, software must be made available to editors and reviewers. We strongly encourage code deposition in a community repository (e.g. GitHub). See the Nature Portfolio guidelines for submitting code & software for further information.

Data

Policy information about availability of data

All manuscripts must include a data availability statement. This statement should provide the following information, where applicable:

- Accession codes, unique identifiers, or web links for publicly available datasets
- A description of any restrictions on data availability
- For clinical datasets or third party data, please ensure that the statement adheres to our policy

The RNA-seq data generated in this study have been deposited in the DNA Data Bank of Japan under accession number DRA015097. Source Data are provided with this paper. All other data supporting the findings of this study are available within the article and its Supplementary Information.

Research involving human participants, their data, or biological material

Policy information about studies with human participants or human data. See also policy information about sex, gender (identity/presentation), and sexual orientation and race, ethnicity and racism.

Reporting on sex and gender	Sex was recorded for the clinical materials used in this study and is reported in Supplementary Figs. 8a and 9a. Gender information was not available to the authors. No sex- or gender-based analysis was performed because the study was not designed or powered for such comparisons.
Reporting on race, ethnicity, or other socially relevant groupings	Race, ethnicity or other socially relevant were not considered in the study design.
Population characteristics	Population characteristics were not considered in the study design.
Recruitment	Hospitalized patients with HHV-6A or HHV-6B genome detected in their blood were recruited. Participants received no compensation.
Ethics oversight	The Ethics Committee of Kobe University Graduate School of Medicine and the Ethical Review Board of Human Studies at Fujita Health University.

Note that full information on the approval of the study protocol must also be provided in the manuscript.

Field-specific reporting

Please select the one below that is the best fit for your research. If you are not sure, read the appropriate sections before making your selection.

☒ Life sciences ☐ Behavioural & social sciences ☐ Ecological, evolutionary & environmental sciences

For a reference copy of the document with all sections, see nature.com/documents/nr-reporting-summary-flat.pdf

Life sciences study design

All studies must disclose on these points even when the disclosure is negative.

Sample size	Sample size calculation was not performed. Sample sizes were chosen based on standard practice in the field, previous experience, sample availability, and the need to obtain reproducible results across independent experiments. The sample size and the definition of n for each experiment are provided in the corresponding figure legends.
Data exclusions	No data were excluded from the analyses.
Replication	Replication was assessed through independent experiments, as indicated in the corresponding figure legends. All attempts at replication were successful.
Randomization	Samples were allocated into experimental groups based on predefined experimental conditions, such as cell type, viral strain, plasmid construct, shRNA or sgRNA expression, or treatment. Random allocation was not used because group assignment was determined by the experimental design and randomization was not relevant to this study.
Blinding	Investigators were not blinded to group allocation during data collection or analysis, as blinding was not considered relevant for this study based on the predefined experimental conditions and objective readouts.

Reporting for specific materials, systems and methods

We require information from authors about some types of materials, experimental systems and methods used in many studies. Here, indicate whether each material, system or method listed is relevant to your study. If you are not sure if a list item applies to your research, read the appropriate section before selecting a response.

Materials & experimental systems

n/a	Involved in the study
[ ]	[x] Antibodies
[ ]	[x] Eukaryotic cell lines
[x]	[ ] Palaeontology and archaeology
[ ]	[x] Animals and other organisms
[ ]	[x] Clinical data
[x]	[ ] Dual use research of concern
[x]	[ ] Plants

Methods

n/a	Involved in the study
[x]	[ ] ChIP-seq
[x]	[ ] Flow cytometry
[x]	[ ] MRI-based neuroimaging

Antibodies

Antibodies used

1. mouse mAb against Flag (Sigma-Aldrich, cat. no. F3165, clone M2, 1:1000)
2. mouse mAb against HA (Sigma-Aldrich, cat. no. H9658, clone HA-7, 1:1000)
3. α-tubulin (Sigma-Aldrich, cat. no. CP06, clone DM1A, 1:1000)
4. mouse mAb against LAMP1 (Santa Cruz Biotechnology, cat. no. sc-20011, clone H4A3, 1:200)
5. mouse mAb against Golgin-97 (Thermo Fisher Scientific, cat. no. 14-9767-82, clone CDF4, 1:200)
6. rabbit mAb against APOBEC3B (5210-87-1342, 1:500)
7. rabbit pAb against GFP (Abcam, cat. no. ab290, 1:1000)
8. rabbit pAb against Flag (MBL, cat. no. PM020, 1:200)
9. rabbit pAb against APOBEC3C (GeneTex, cat. no. GTX102164, 1:500)
10. rabbit pAb against Calnexin (Abcam, cat. no. ab22595, 1:500)
11. rabbit pAb against LC3B (Abcam, cat. no. ab51520, 1:500).
12. mouse mAb to HHV-6A IE2 (AIE2-1)
13. mouse mAb to U14 (BU14)
14. mouse mAb to gB (OHV-1)
15. mouse mAb to gQ1 (AgQ1-119)

Validation

1. https://www.sigmaaldrich.com/JP/en/product/sigma/f3165
2. https://www.sigmaaldrich.com/JP/en/product/sigma/h9658
3. https://www.merckmillipore.com/JP/en/product/Anti-Tubulin-Antibody-clone-DM1A,MM_NF-MABT205
4. https://www.scbt.com/p/lamp-1-antibody-h4a3
5. https://www.thermofisher.com/antibody/product/Golgin-97-Antibody-clone-CDF4-Monoclonal/14-9767-82
6. Cancer Res 75, 4538-4547, doi:10.1158/0008-5472.CAN-15-2171-T (2015).
7. https://www.abcam.co.jp/products/primary-antibodies/gfp-antibody-ab290.html
8. https://ruo.mbl.co.jp/bio/dtl/A/?pcd=PM020
9. https://www.genetex.com/Product/Detail/APOBEC3C-antibody/GTX102164
10. https://www.abcam.co.jp/products/primary-antibodies/calnexin-antibody-er-marker-ab22595.html
11. https://www.abcam.co.jp/products/primary-antibodies/lc3b-antibody-ab51520.html
12. J Virol 91, doi:10.1128/JVI.01121-17 (2017).
13. J Virol 79, 13037-13046, doi:10.1128/JVI.79.20.13037-13046.2005 (2005).
14. J Virol 77, 4992-4999, doi:10.1128/jvi.77.8.4992-4999.2003 (2003).
15. J Virol 77, 2452-2458, doi:10.1128/jvi.77.4.2452-2458.2003 (2003).

Eukaryotic cell lines

Policy information about cell lines and Sex and Gender in Research

Cell line source(s)

MT4, HSB2, JJhan, and HEK293T cells were maintained in the laboratory of Yasuko Mori. Primary CBMCs were purchased from the Cell Bank of the RIKEN BioResource Center, Tsukuba, Japan. The sex of the CBMC donors was not recorded.

Authentication

The human cell lines used in this study were checked against the current ICLAC Register of Misidentified Cell Lines, and none were listed. The cell lines were not further authenticated by the authors after receipt.

Mycoplasma contamination

The cell lines were not tested for mycoplasma contamination.

Commonly misidentified lines (See ICLAC register)

Not applicable.

Animals and other research organisms

Policy information about studies involving animals; ARRIVE guidelines recommended for reporting animal research, and Sex and Gender in Research

Laboratory animals	Immunodeficient 129S4-Rag2tm1.1Flv Il2rgtm1.1Flv Tg(SIRPA)1Flv/J (hSIRPα-DKO) mice and 129S4-Rag2tm1.1Flv Il2rgtm1.1Flv/J (DKO) mice were maintained. Four months old mice were used.
Wild animals	The study did not involved wild animals.
Reporting on sex	Sex was not considered.
Field-collected samples	The study did not involved samples collected from the field.
Ethics oversight	The Institutional Animal Care and Use Committee of Kobe University.

Note that full information on the approval of the study protocol must also be provided in the manuscript.

Clinical data

Policy information about clinical studies

All manuscripts should comply with the ICMJE guidelines for publication of clinical research and a completed CONSORT checklist must be included with all submissions.

Clinical trial registration	Not applicable.
Study protocol	Not applicable.
Data collection	Not applicable.
Outcomes	Not applicable.

Plants

Seed stocks	Not applicable.
Novel plant genotypes	Not applicable.
Authentication	Not applicable.
